# Standardization of QRS Duration Measurement and LBBB Criteria in CRT Trials and Clinical Practice

**DOI:** 10.2174/157340313805076269

**Published:** 2013-02

**Authors:** Mohit K Turagam, Poonam Velagapudi, Abraham G Kocheril

**Affiliations:** 1 Department of Medicine, University of Wisconsin School of Medicine and Public Health, 3116 MFCB, 1685 Highland Avenue, Madison, WI-53705, USA; 2 Division of Electrophysiology and Cardiovascular Medicine, Department of Medicine, University of Illinois College of Medicine Urbana Champaign, 611 W Park St, Urbana, IL 61801

**Keywords:** Cardiac resynchronization therapy, CRT non responders, LBBB criteria, QRS duration.

## Abstract

Based on the clinical trials so far, there is a major controversy regarding the benefit of CRT in patients with QRS≤150 milliseconds. Some studies have shown that a fair number of patients with QRS ≤150 milliseconds benefit from CRT and it is needless to say that careful attention should be paid to CRT non-responders considering the risk of complications and cost-benefit ratio. Lack of uniformity in QRS measurement in all these trials could have a major influence on variable study outcomes. This is of concern because when the QRS is close to 120 milliseconds in patients with NYHA class III/IV symptoms or QRS close to 150 milliseconds in NYHA class I/II patients, the decision to recommend CRT implantation or undertake further risk stratification investigations is critically dependent on the EKG interpretation. In this paper we intent to raise the important question for need of standardized electrocardiographic criteria (QRS measurement and LBBB) in patients enrolled in CRT trials considering the variability in study results, high rates of CRT non response in the eligible population and the associated health care cost burden.

## INTRODUCTION

The American College of Cardiology (ACC), American Heart Association (AHA), Heart Rhythm Society (HRS), European Society of Cardiology (ESC), and Heart Failure Society of America (HFSA) recommended cardiac resynchronization therapy (CRT) as a class I indication in patients with New York Heart Association (NYHA) class III-IV congestive heart failure (CHF) with an EF ≤35% and QRS duration of ≥120 milliseconds, these guidelines were based on the results of two major prospective randomized clinical trials [1,2]. CRT has been shown to improve mortality, heart failure hospitalizations, exercise performance, peak oxygen consumption and overall quality of life in patients with New York Heart Association (NYHA) class III-IV CHF [1,2]. Recently, the HFSA [3] and ESC [4] guidelines for management of CHF were revised in response to the Multicenter Automatic Defibrillator Implantation Trial With Cardiac Resynchronization (MADIT-CRT) [5] and the extended follow-up of the Resynchronization Reverses Remodeling in Systolic Left Ventricular Dysfunction (REVERSE) [6] sub study. These new guidelines introduced a new recommendation for CRT in NYHA class I and/or II systolic CHF, but this time with a new QRS cutoff of ≥150 milliseconds for this population. A recent meta-analysis by Sipahi *et al.* including five randomized trials (COMPANION, CARE HF, MADIT CRT, REVERSE and RAFT) reported that CRT did not reduce events (death, hospitalization) in patients with QRS ≤150 milliseconds (RR, 0.95 [95% CI, 0.82-1.10]) (p = 0.49) [7]; raising several important questions regarding CRT eligibility in the general population as well as in clinical trials. The problem of non-response to CRT is becoming increasingly important as approximately 40% of CRT devices are now implanted in patients with a QRS duration of ≤150 milliseconds [8] and with the revision of new HFSA and ESC guidelines extending the recommendation of CRT in NYHA class I and II heart failure patients; increasing the number of CRT eligible patients in the following years. There is a general consensus that most patients with QRS≥150 milliseconds benefit from CRT. However there is major controversy regarding the benefit of CRT in patients with QRS≤150 milliseconds. Studies have shown that a fair number of patients with QRS ≤150 milliseconds benefit from CRT and it is needless to say that careful attention should be paid to CRT non-responders considering the risk of complications and cost-benefit ratio. In this review we intent to raise the important question for need of standardized electrocardiographic criteria (QRS measurement and LBBB) in patients enrolled in CRT trials considering the variability in study results, high rates of CRT non response in the eligible population and the associated health care cost burden. 

## CRT NON-RESPONSE

What is “CRT non-response”? CRT non-response can be broadly defined as inability to significantly impact patient’s symptoms, improve quality of life and possibly affect outcomes in CRT eligible patients. The rate of non-response to CRT is often quoted to be about 30%. Quantifying CRT non-response can be complex and depends on the outcome measure chosen such as symptom improvement, quality of life, heart failure hospitalizations, left ventricular remodeling and mortality. Assessment of CRT non-response can be difficult as the desired benefit from CRT cannot be generalized and depends on the individual patient’s overall condition, underlying etiology of heart disease, functional capacity, genetic ability and patient expectation. 

## LACK OF STANDARDIZATION

The major randomized clinical trials^1,2^ that led to the widespread adoption of CRT used a QRS duration of 120 milliseconds but did not select patients on the basis of QRS morphology. In comparison PATH-CHF II [9], COMPANION [1], CARE-HF [2] the Cardiac Resynchronization Therapy in Patients with Heart Failure and Narrow QRS (RethinQ) [10], MADIT-CRT [5], REVERSE [6] and the most recent Resynchronization – Defibrillation for Ambulatory Heart Failure Trial (RAFT) [11] study all consistently showed that CRT was progressively less effective in reducing frequency of hospitalization, and death rate as QRS duration decreased. However, the specific methods for QRS measurement were not described in these publications, and international guidelines do not specify a preferred/standard measurement technique [3,4]. Lack of uniformity in QRS measurement in all these trials could have a major influence on variable study outcomes. This is of concern because when the QRS is close to 120 milliseconds in patients with NYHA class III/IV symptoms or QRS close to 150 milliseconds in NYHA class I/II patients, the decision to recommend CRT implantation or undertake further risk stratification investigations is critically dependent on the EKG interpretation. Thus, inaccurate QRS classification may increase costs by exposing patients to otherwise unnecessary invasive procedures or deny some patients the beneficial effects of CRT. 

## MEASURING QRS DURATION

QRS duration is measured from the beginning of the Q wave to the end of the S wave. A normal range is from 40 to 100 milliseconds (1 small box to 2.5 small boxes). Modern EKG equipment permits the EKG recordings to be obtained in a digital format and stored in a computer readable form. In some centers, especially the small and unspecialized units, short-term EKGs are still recorded and kept on paper. Short-term EKGs have been collected as paper recordings also in many multi center and other extensive studies. Hence, a significant number of resting 12-lead EKGs exist in paper format which may contain a significant amount of information that may be useful for researching and outcome analysis in clinical studies. Most frequently, manual measurements are performed directly on the paper recorded tracings. These paper recordings impose serious restrictions on the possibility of a detailed analysis of EKG patterns, especially QRS duration and forms. This concerns particularly those measurements involving very small sections of the EKG, the correct identification of which can be seriously affected by tiny absolute errors. Several studies have been conducted showing the difficulties in measuring QRS duration and QT dispersion [12]. Data obtained with manual calipers are known to be biased by preferential numbers and the overall precision of manual calipers is not impressive. With calipers, it is particularly difficult to obtain a precise value of an interval when the baseline of the tracing is distorted. High precision digitizing boards with resolutions between 10 and 100 micrometers have been accepted as the gold standard technology for manual measurements of paper EKG. While it seems obvious that localizing the same point repeatedly with a precision of ± 10 micrometers is beyond human reach, no data presently exist on the manual precision of operating a digitizing board, including the manual measurement of EKGs. Malik *et al.*, 1997 [13] investigated the precision achieved by human measurement on a digitizing board and concluded that human precision of operating a digitizing board is rather poor. In consequence digital EKG tracings and on-screen calipers have replaced paper EKG printouts and digitizing board as the primary tools for EKG acquisition and interval measurement in clinical trials. The only written recommendations for EKG interval measurement widely accepted before the digital era were published in 1997 by the European Committee for Proprietary Medicinal Products and were based on annotating 3 consecutive sinus complex, preferably from lead II [14]. The introduction of on-screen methodologies based on digital EKGs has completely changed the measuring environment. For example, the potential advantages of implementing digital algorithms are now being considered. Consequently, pharmaceutical sponsors commonly use semi automated methods for centralized EKG interval measurement, where a trained human analyst decides whether the EKG interval annotations by the automated algorithm should be adjusted based on visual inspection of annotated waveforms on a computer screen. This approach potentially combines consistency of the automated interval measurement with the added precision of manual adjustment, although no data have been published thus far on the performance of semi automated method.

An equally important aspect which has not received importance is to where to measure QRS duration. Due to the absence of new guidelines there is lack of uniformity in EKG interval measurement in majority of studies being carried out. A study by Hamlein *et al.* [15] investigated the number of cardiac cycles that must be measured from a dog to accurately characterize the relationship between RR and QT intervals. In this study, EKGs’ were obtained from 12 conscious dogs with sinus rhythm. In each dog, RR and QT intervals were measured for 12 cardiac cycles. Measurements for each were then averaged over all 12 cycles, and those results compared to the average of both the initial 6 and 3 cycles, as well as to the middle cycle alone, for 12, 6, and 4 of the dogs. The study reported that there was no significant difference in the results of measurements of RR, QT, or QTc obtained from 12, 6, 3, or 1 cycle, whether from 12, 6, or 4 dogs. Intraobserver variability of EKG measurements was tested by having a single observer measure 10 copies of 12 different EKGs. The greatest coefficient of variation for the measurement of any EKG parameter was less than 2.5%. The study concluded that measurements of RR and QT intervals made by a trained observer from 1 cardiac cycle accurately reflect those that are averaged from 3, 6, or 12 cycles whether the number of dogs per group is 12, 6, or 4. Manual QRS duration assessments demonstrate significant variability, and concordance with computer-calculated measurement depends on whether QRS duration is defined as the mean or maximal value. Tomlinson *et al.*, 2009 [16] investigated the effect of EKG display format and paper speed on the accuracy of manual QRS duration assessment and concordance of manual QRS duration with computer-calculated mean and maximal QRS duration. Six cardiologists undertook QRS duration measurements on EKGs, with computer-calculated mean QRS duration close to 120 milliseconds. Display formats were 12-lead, 6-limb, and 6-precordial leads, each at 25 and 50 mm/s. When the computer-calculated mean was used to define QRS duration, manual assessment demonstrated 97 and 83% concordance at categorizing QRS duration as and >120 ms respectively. Using the computer-calculated maximal QRS duration, manual assessment demonstrated 83% concordance when QRS duration was, 120 ms and 19% concordance when QRS duration was >120 ms. The six-precordial lead format demonstrated significantly less intra and inter-observer variability than the 12-lead, but this did not improve concordance rates. The study concluded that manual QRS duration assessments demonstrate significant variability, and concordance with computer-calculated measurement depends on whether QRS duration is defined as the mean or maximal value. Consensus is required both on the most appropriate definition of QRS duration and its measurement. Finally, the lack of a clear guideline on methods for QRS measurement may create intra- and inter-individual inconsistencies in device prescription.

## CRT AND LBBB

Sweeny *et al.* demonstrated that an EKG pattern for complete left bundle branch block (LBBB) is a strong predictor of response to CRT [17]. This observation was consistent with the recent COMPANION [1], MADIT-CRT [5] and RAFT [11] study. In the COMPANION trial, patients without LBBB did not have a statistically significant benefit, and those with QRS durations ≤147 milliseconds had absolutely no benefit^1^. MADIT-CRT [5] trial enrolled patients with NYHA class I and II CHF and QRS durations ≥130 milliseconds. The presence or effect of LBBB versus right bundle branch block (RBBB) was not reported in the original publication; however, patients with QRS durations ≤150 milliseconds received no benefit. Subsequent analysis showed that patients with LBBB receiving CRT had a very significant 55% decrease (hazard ratio [HR] 0.45, p <0.05) in CHF hospitalizations or mortality, while patients without LBBB had a non–statistically significant increase in these adverse events [18]. Additional analysis also considered thresholds of QRS duration separately for men and women. The benefit from CRT was highly significant in women beginning at a QRS duration ≥130 milliseconds (QRS duration 130 to 139 ms HR 0.19, p <0.05), but there was no benefit in men with QRS durations ≤140 ms (HR 1.12, p >0.05) and a non-significant benefit in the group with QRS durations from 140 to 159 ms (HR 0.76, p >0.05). This was supported by another study in 14,946 Medicare patients with NYHA Class III/IV receiving CRT showed that those without LBBB had significantly increased mortality compared to patients with LBBB, and QRS duration 150 milliseconds predicted more favorable outcomes in LBBB but not in RBBB [19].In comparison clinical data from the MADIT-CRT [5] and RAFT trial [11], which indicate that in CRT patients with RBBB, there was a marginal reduction in the number of hospitalizations but an higher risk of death, or major arrhythmic events compared to the control group. Interestingly, CRT patients with diffuse intraventricular conduction disturbances showed an increased risk for hospitalization, and a near 2-fold higher risk of death compared to ICD patients. 

Therefore, while initially QRS duration was the selection criterion (and still is in the guidelines), it seems that a LBBB configuration should be the most important criterion. A recent study by Seo *et al.* [20] reported that complete LBBB was identified as an independent predictor of CRT responders and more strongly associated with the endpoint (hazard ratio, 0.38, 95% confidence interval 0.20-0.72, P = 0.003) than any of the echocardiographic parameters. Another metanalysis by Sipahi *et al.* [21] including four randomized trials totaling 5,356 patients with LBBB at baseline demonstrated that there was a highly significant reduction in composite adverse clinical events with CRT (RR = 0.64 [95% CI (0.52-0.77)], P = .00001). The study also reported that there was no benefit for patients with non-LBBB conduction abnormalities (RR = 0.97 [95% CI (0.82-025; CI (0.82-1.15)], P = .75) and that there was a significant difference in effect of CRT between LBBB versus non-LBBB (P = .0001). Though there is emerging data to say that complete LBBB may be used as criteria for CRT placement, there are some fundamental problems regarding criteria for defining complete LBBB. Similarly another recent study by Perrin *et al.* demonstrated that in patients with guideline-defined LBBB, the absence of EKG markers of residual left bundle conduction was predictive of a greater response with CRT [22]. According to the ACC/AHA/HRS recommendations regarding criteria for LBBB include QRS duration ≥120 ms, QS or rS in lead V1, monophasic R wave with no Q waves in leads V6 and I, broad notched or slurred R wave in leads I, aVL, V5 and V6 and an occasional RS pattern in V5 and V6 [23]. There are three clinical studies suggesting that a third patients diagnosed with complete LBBB by EKG crtieria may not tryly have one. [24,25,26]. Strauss *et al.* [27] recently proposed a new criteria for complete LBBB which included QRS duration ≥140 ms (men) or 130 ms (women), QS or rS in leads V1 and V2, and mid-QRS notching or slurring in ≥2 of leads V1, V2, V5, V6, I, and aVL. 

## CONCLUSION

We propose that a consensus view needs to be established on how best to measure QRS duration (manual vs. computerized); if computerized standardization on the company manufacturing the EKG machine is not in clinical practice but in trials; whether to use the mean or maximal QRS duration also reconsider the diagnostic criteria for LBBB to better standardize decision-making in CRT implantation.
